# Leveraging Lifestyle Medicine Interest Groups Through the COVID-19 Pandemic

**DOI:** 10.1177/1559827620936595

**Published:** 2021-03

**Authors:** Lisa Kisling Thompson, Tatiana Znayenko-Miller, Daniel Gorenstin, Krystyna Rastorguieva, Sami Bég, Elizabeth Frates, Beth Frates

**Affiliations:** University of Colorado Denver, Anschutz Medical Campus, Aurora, Colorado (LKS); The George Washington University School of Medicine and Health Sciences, Washington, DC (TZM); Universidade Federal do Estado do Rio de Janeiro, Rio de Janeiro, Brazil (DG); Rollins School of Public Health, Emory University, Atlanta, Georgia (KR); Proactive Living, Inc, Columbia, South Carolina (SB); Harvard Medical School, Spaulding Rehabilitation Hospital, Charlestown, Massachusetts (EF, BF)

**Keywords:** interest group, lifestyle medicine, LMIG, trainees

## Abstract

Lifestyle medicine domains, despite accounting for more than 78% of chronic disease risk, are infrequently taught as a part of the medical curriculum. Aspects such as nutrition are taught in less than 25% of medical schools, a statistic that continues to decline, and less than 20% of practicing physicians were required to take even a single course in exercise counseling during their medical school training. To combat this lack of training, the American College of Lifestyle Medicine annually awards the Donald A. Pegg scholarship to fund the development of Lifestyle Medicine Interest Groups (LMIGs) across medical schools worldwide. This scholarship was initiated in 2016 and utilizes private funds to support the development and expansion of LMIGs with the aim of increasing awareness of lifestyle medicine among training practitioners. There are four award winners per year. To date there are sixteen Pegg Award winners. This article will showcase the four 2019-2020 Donald A. Pegg award recipients and their impact on the LMIGs at their institutions. Furthermore, it highlights the ingenuity and adaptation of these LMIGs during the COVID-19 pandemic.

‘Lifestyle medicine domains, despite accounting for more than 78% of chronic disease risk, are infrequently taught as a part of the medical curriculum.’

Lifestyle medicine is defined as “The evidence-based practice of helping individuals and families adopt and sustain healthy behaviors that affect health and quality of life.”^1^ This definition, along with this innovative field of medicine, was developed in 2010 through the *JAMA* article “Physician Competencies for Prescribing Lifestyle Medicine.”^1^ The clinical practice of lifestyle medicine centers primarily on the promotion of a plant-based diet, physical activity, healthy sleep, stress management, reduction and/or cessation of risky substance use, and strengthening of social support. Utilization of these key elements allows for lifestyle medicine practitioners to effectively and sustainably reverse and prevent chronic diseases such as type 2 diabetes mellitus, hypertension, dyslipidemia, and more.^2^

Lifestyle medicine domains, despite accounting for more than 78% of chronic disease risk, are infrequently taught as a part of the medical curriculum.^3^ Aspects such as nutrition are taught in less than 25% of medical schools, a statistic that continues to decline, and less than 20% of practicing physicians were required to take even a single course in exercise counseling during their medical school training.^2,4^

To combat this lack of training, the American College of Lifestyle Medicine (ACLM) awards the Donald A. Pegg scholarship which funds the development of Lifestyle Medicine Interest Groups (LMIGs) across medical schools worldwide. This scholarship was initiated in 2016 and utilizes private funds to support the development or expansion of LMIGs in an effort to increase awareness of lifestyle medicine among training practitioners. Furthermore, the scholarship provides travel funds for recipients to attend the ACLM’s annual conference where their knowledge about lifestyle medicine as well as their international network are strengthened. There are four award winners per year. To date there are sixteen Pegg Award winners.

## Introduction

Interest groups represent a unique opportunity to expand awareness, training, and education in the university environment, using relatively few resources. These groups are often organized and facilitated by students with minimal faculty oversight and encourage collaboration, academic development, and leadership among trainees with common interests. With these goals in mind, Harvard Medical School developed the first LMIG in 2009, which has since served as a model for the establishment of new LMIGs throughout universities worldwide.^5^ As the field of lifestyle medicine continues to grow, the LMIG presents as a malleable and vital resource for the education and empowerment of future health care practitioners.

This article will showcase the four 2019-2020 Donald A. Pegg award recipients and their impact on the LMIGs at their institutions. Furthermore, it highlights the ingenuity and adaptation of LMIGs during the COVID-19 pandemic.

## Pegg Award Winner Reports

### Daniel Gorenstin, Medical Student, Universidade Federal do Estado do Rio de Janeiro, Founder of the LMIG

In the last few decades, the role of the medical school in the students’ training has been reframed. Today, as there are numerous ways of acquiring medical knowledge, one of the new roles of the medical school is emphasizing what is most important for the professional lives of the future physicians. As such, the implementation of lifestyle medicine as part of the core curriculum is imperative, seeing as it is extremely useful to physicians and present in nearly all specialties. The surge of LMIGs in the United States and around the world is happening, in part, because students understand this new dynamic and take it upon themselves to fill this gap, while the vast majority of medical schools still lack a course in lifestyle medicine—showing they are not yet in the action stage of change.

As a medical student in Brazil—learning at a university hospital that is part of the public health system—I quickly understood that it would be my generation’s duty to design strategies aimed at improving national health care. With 75% of Brazil’s population entirely dependent on the universal tax-funded health system, the main issues related to the imbalance of health care demand and provision stem from lack of capital.^6^ Therefore, Brazil is very much in need of solutions that contribute in terms of disease prevention and cost-effective, first-line treatments sparing hospitalization and unnecessary use of drugs.

When I first heard of lifestyle medicine at a Semiology class by Prof Lucas Medeiros, MD—who is now the LMIG’s faculty advisor—it was easy to put things together in my mind, and I understood the importance as well as the potential impact of lifestyle medicine.

Since starting our LMIG in early 2019, we have developed many activities—always looking to bring in International Board of Lifestyle Medicine–certified physicians—such as lectures of all kinds and talks in the “Questions and Answers” format in which students can freely discuss aspects of lifestyle medicine with specialists in the field. We are currently in the process of initiating culinary medicine activities for this school year, in an effort to bring information regarding the benefits of whole-food, plant-based diets and at the same time create a more dynamic and interesting way of learning. Having the opportunity to apply for Taste of Lifestyle Medicine micro-grants through the ACLM ensures that we will be able to bring culinary medicine experiences to our students.

Currently, around 15 to 25 students attend each activity, which is more than what is usually seen in interest groups on our campus. In our first year, our main goal was to engage the students as much as possible and transmit to them the importance of continuing to involve future students, in order to assure the continuation of the LMIG after its founders have graduated—which is of the utmost importance.

The COVID-19 pandemic and its quarantine came right after the summer break in the Southern Hemisphere. This has definitely imposed barriers, due to the impossibility of in-person activities, such as the new culinary medicine activities we had planned. However, we remain just as motivated and will work to make those types of activities a success as soon as the health situation allows. Meanwhile, our meetings are taking place online so that we can keep moving toward our goal. We always bear in mind our important mission to promote lifestyle medicine in our medical school.

With the grant given by the Donald A. Pegg Award, we now have the funding to develop our LMIG further. We have the possibility of providing healthy snacks for regular activities that last longer and of acquiring material for the culinary medicine activities that we are planning for this year. Also, we can refrain from charging fees that discourage student attendance.

Our LMIG is grateful to the ACLM for supporting future health professionals in their lifestyle medicine endeavors. I believe an award attests to the quality and effort of the team of people who have donated their time to the cause in the interest of improving medical training for students and making lifestyle medicine more present in the minds of future physicians and other health professionals. The Donald A. Pegg Award has definitely changed our LMIG and the visibility this award has brought has undoubtedly sparked more interest among students who have never attended an activity. The future of the LMIG at our medical school is promising, and we are extremely thankful for all the help.

### Krystyna Rastorguieva, Executive Master of Public Health Student, Prevention Science Track, Rollins School of Public Health at Emory University, Atlanta, GA, and Founder and President of Emory Lifestyle Medicine Interest Group

Before the foundation of Emory Lifestyle Medicine Interest Group in the fall of 2019, lifestyle medicine efforts at Emory University were rather disjointed. Emory did have an LMIG that existed here some years ago, but it since has dissipated and no active members were on campus any longer.

My personal journey leading to joining ACLM and founding an LMIG was somewhat untraditional. I entered the workforce at Emory Healthcare almost 4 years ago. In parallel, on my own time, I was putting time and effort into educating myself on the power of plant-based nutrition inspired by my then recent transition to a vegan diet. I have attended several plant-based nutrition conferences (Remedy Food Atlanta 2016, Plant-Based Prevention of Disease [P-POD] 2017) and after realizing the power and potential for integrating this knowledge in health care decided to enter the Executive Master of Public Health (MPH) program at Emory to gain the tools and knowledge needed to make a meaningful impact. I quickly learned about ACLM and joined as well.

On my journey I have built a network of like-minded and motivated individuals at Emory who encouraged me to apply for the Donald A. Pegg Award and help further galvanize lifestyle medicine efforts at Emory. I was lucky enough to have the honor to receive the Donald A. Pegg grant at ACLM 2019, which gave the Emory LMIG the credibility and support needed to start the work, gain visibility, and create a platform for students, faculty, and practicing health care professionals to unite around the idea of lifestyle medicine. Sharon Bergquist, MD, Assistant Professor at Emory School of Medicine, Medical Director of Emory Lifestyle Medicine and Wellness, and a practicing primary care physician, became our faculty adviser.

We started with the Kick-Off event on December 4, where Jonathan Bonnet, MD, presented about lifestyle medicine. We worked with our hospital Chef, Mike Bacha, to provide a beautiful plant-based spread and had 27 people join in person for the presentation and an engaging discussion after.

Since then we have hosted 3 events (every 1 to 2 months), with average in-person participation of 25 to 30 people. Events included a working session to brainstorm and clarify Emory LMIG vision; a presentation and social gathering on the topic of “The Power of Plant-Based Nutrition” with Arvinpal Singh, MD, who is an ACLM board certified physician and a medical director of Emory Bariatric Center; and a screening of the documentary *Resilience* followed by a discussion with Community Resiliency Model certified practitioner Linda Grabbe, PhD. We had a full schedule of monthly events planned through June (including a workshop on 21-Day Plant-Based Diet Challenge with partners at Kaiser; Volunteer day at Farm Sanctuary; Code Blue documentary screening), touching on different aspects of lifestyle medicine and partnering with other groups at Emory University and beyond. Our culminating event was supposed to be a talk with Michael Klaper, MD, on “Moving Medicine Forward.”

COVID-19 forced us to alter our plans and shift the focus and format of our efforts. With many of our core group members being closely involved on the front lines of the pandemic, some of the group activity has been temporarily paused. At this point we are reworking our schedule and are planning a number of virtual events for upcoming months (Journal study, Exercise as Medicine interactive webinar, and other events).

We have managed to establish, and plan to continue to develop, valuable relationships with various partners at Emory University, Emory Healthcare, and the community overall. The intention and unique trait of Emory LMIG is the multidisciplinary aspect of it—our hope is to unite students and health care professionals from School of Medicine, School of Nursing, School of Public Health, and Business and Law Schools to create a synergetic and multifaceted community of problem-solvers passionate about lifestyle medicine. So far, we have found key contacts at each of the schools and will work to keep each other engaged with collaboration opportunities. We have also established a close partnership with Emory University Hospital Nutrition Services team, and specifically Executive Chef Mike Bacha, who has diligently worked with us to provide locally sourced vegan and plant-based options for our events. Another partner is Emory Office of Sustainability, which provided guidance and support to make the Emory LMIG event zero waste.

The Donald A. Pegg grant; personal support, guidance, and encouragement from Beth Frates, MD, and Sami Beg, MD; and help available from ACLM have served as both the resource and motivation to make this work possible.

### Lisa Kisling Thompson, PGY-3, University of Colorado, Resident Founder of LMIG

Throughout medical school and even two years into residency training, I remained frustrated with the traditional medical curriculum: one that focuses on disease treatment rather than health promotion. I felt that I continually put Band-Aids on my patients’ issues, only to have them return weeks later with the same cut rearing its angry head again. It was not until I began my personal search for effective medicine that I found the antidote to the underlying issue: lifestyle medicine. This practice, dedicated to the promotion of health, sat in stark contrast to my current understanding of symptomatic disease treatment. I immediately fell in love with the concept and retargeted my career to become a lifestyle medicine practitioner.

As a preventive medicine resident, I was awarded the unique opportunity of focusing on prevention. Unfortunately, opportunities for lifestyle-based preventive measures still felt out of reach. Despite the robust medical community in the Denver metro area, including at the University of Colorado’s medical campus, lifestyle medicine training opportunities were rare. The close-knit lifestyle medicine practitioner community sponsored seminars when prompted, but otherwise training remained minimal. My passion for this field, in combination with the lack of training in traditional medical settings, spurred me to apply for the Donald A. Pegg scholarship.

This grant will help award medical students, residents, nursing students, and pharmacy professionals an avenue to explore evidence-based and effective treatments for the chronic diseases plaguing our community. While formally developing an LMIG as a resident remains challenging, we are developing the infrastructure for a robust and successful group. Currently, we have outlined six bimonthly meeting agendas interspersed with evidence-based resources and surveys for data tracking. The grant money from the Pegg scholarship is allowing our leadership the opportunity to develop a culinary medicine seminar where participants will cook and enjoy a plant-based meal in the Anschutz Health and Wellness Center’s commercial kitchen. Additionally, it is providing funding for plant-based refreshments at the other five meetings where participants will interact with like-minded practitioners, learn business skills for practicing lifestyle medicine, and work with the local Walk with a Doc chapter.

While I was beyond excited for these innovative in-person meetings, the development of the COVID-19 pandemic in early 2020 prompted a need for innovation and a change of plans. In preparation and response to the pandemic, we shifted our LMIG to a virtual platform. While these changes were unprecedented, we are excited to develop a webpage for the University of Colorado’s LMIG where seminars, talks, and resources for interested students will be housed. Additionally, we hope to develop support groups that can meet virtually to promote wellness through and after the pandemic. As everyone learns to adapt to a new normal, we are confident our lifestyle medicine practices will flourish in helping students, residents, and patients alike in doing so.

While development of the formal interest group remains a challenge, awareness of the practice of lifestyle medicine continues to grow across our campus. We have piloted a lifestyle medicine residency training track that has been made available to preventive, family, and internal medicine residents and allows for dual board certification upon completion of the track. As educational opportunities such as this continue to develop, interest in the field ignites and spreads. The University of Colorado’s LMIG looks forward to creating a robust continuing education curriculum offered virtually, and hopefully in-person in the future. We are grateful to have the funding of the Pegg grant to help us continue to expand awareness of lifestyle medicine!

### Tatiana Znayenko-Miller, GWU, Master of Science in Integrative Medicine at The George Washington University, Medical Student (Matriculating Fall 2020)/Founder of the LMIG at GWU

I grew up in a region of North Carolina that was—and is—devastated by chronic disease.^7^ It was commonplace for the members of my community to communicate that many of these chronic diseases (cancer, metabolic syndrome, type 2 diabetes) were “inevitable,” or simply the “reality of aging.” I embraced this understanding of chronic disease as insoluble and carried it with me into the beginning of undergraduate experience at The George Washington University (GWU). At GWU, I began to engage with courses, faculty members, and clinical experiences that motivated me to look more closely at the etiology of chronic disease and the modifiable lifestyle factors—nutrition, exercise, stress, social support, and sleep—that could fundamentally affect the trajectory of chronic disease.^8^ I have since elected to dedicate my medical career to the study, practice, and perpetuation of this knowledge, formally termed “lifestyle medicine.”^9^

The Donald Anderson Pegg Student Leadership Award has buttressed my passion for lifestyle medicine, and generated an avenue through which I and other students of medicine, health sciences, and public health, faculty members, and health care professionals have been able to mobilize the strengths of the GWU learning environment to enhance the opportunities available to learn about and experience lifestyle medicine. GWU, located in Washington, DC, maintains an enrollment of 6054 undergraduate, graduate, and certificate candidate students across the School of Medicine and Health Sciences, School of Public Health, and School of Nursing.^10^ GWU’s proximity to innovative health care delivery systems (The George Washington University Hospital; Children’s National Medical Center; Sibley Memorial Hospital) and public health institutes (The Centers for Disease Control and Prevention; The National Institutes of Health), in conjunction with its exemplary international faculty and diverse intellectual community, render the university an optimal incubator for thought leadership, collaboration, and academic and professional excellence. The institutional culture of GWU emphasizes these strengths, and delineates the importance of this established framework being mobilized by students to develop and manipulate sustainable solutions to the intractable challenges that affect our nation and world.^11^ The LMIG at GWU is a product of this framework and culture, incepted to function as a platform for the cultivation of service-oriented lifestyle medicine professionals united to address the burden of chronic disease.

Formally established in the Spring of 2020, the LMIG at GWU maintains a student body of approximately 50 undergraduate, graduate, and certificate candidate students organized under the tutelage of faculty advisor Kaylan Baban, MD, Chief Wellness Officer for GWU. The primary objective of the GWU LMIG—to provide opportunities for the acquisition and application of knowledge in the tenets lifestyle medicine—bifurcates to inform the development of the GWU LMIG’s dual, service-oriented objectives: to cultivate health and wellness within the GWU community and throughout the greater communities to which members belong. For this reason, all of the lectures included in the GWU LMIG’s Fall 2020 curriculum, which will feature leaders in the field of lifestyle medicine—Dr James S. Gordon, Dr Joel Kahn, Dr Joel Fuhrman, and Dr Zeeshan Ali—are accessible and free to all student, faculty, and staff members of GWU.

Prior to the emergence of the COVID-19 pandemic, it had been determined that the lectures included in GWU LMIG’s Fall 2020 curriculum would also be webcast to further augment viewership and enable online-only degree students at GWU to benefit from and participate in these lectures. As of May 2020, GWU’s plan for campus reopening during the Fall 2020 has not yet been established, so this previously auxiliary option may be transmuted to function as the primary avenue of delivery. Moreover, as the relationship between chronic disease status and COVID-19 related death and disability becomes more fully delineated, it may be necessary to collaborate with lecturers to enable the modification of content to more appropriately demarcate this connection and provide a sounding board from which GWU’s LMIG cohort can begin to develop accessible, practical, and sustainable solutions to address projected student and community needs.

A project that is, concurrently, underway at the GWU LMIG, with the support of Leigh Frame, PhD, Director of Integrative Medicine at the GWU School of Medicine and Health Sciences, is the development of a multidisciplinary wellness initiative focused on mindfulness-based self-care and group support for the graduate students of the School of Health Sciences at GWU. To date, the scope of this project has included identifying the needs of this population, designing an appropriate curriculum, and planning the implementation of this program to support student emotional and social health and resilience. In the context of the COVID-19 pandemic, a new needs assessment will be conducted during the Summer of 2020, with the intention of more appropriately delineating the unique challenges that students may now be experiencing.^12^

In the interim, the GWU LMIG is actively responding to the stress and trauma of the COVID-19 pandemic by implementing an initiative designed to provide immediate social support to student members.^13^ Through a series of virtual gatherings, the GWU LMIG seeks to restore connectivity and community among members and provide accessible, practical, and evidence-based resources for stress management and reduction. It is the ultimate intention of the GWU LMIG that this initiative be expanded throughout GWU by enabling GWU LMIG members to function as “self-care” peer counselors, thus continuing, even in this time of physical distancing, the service-oriented mission of GWU LMIG.

## Best Practices Summary

- Identify “champions” in varying training levels—medical students, faculty, practicing physicians, residents, and so on—to help promote LMIGs.
- Utilize technology to track metrics on everything—you never know what will be needed in future research or write-ups!
- Identify a technology champion who can help manage social media, webpages, archive presentations, and so on.
- Find partners (other groups in different schools) who share similar interests and partner up on events.
- Gain visibility for an LMIG through newsletters and other social media formats.

## Conclusion

The Donald A. Pegg scholarship has created an atmosphere of education, innovation, and growth among these four campuses. Not only has the scholarship allocated funding to help start and support these much-needed LMIGs, but the recipients have developed a strong, collaborative community of like-minded individuals. Through the wonders of current technology, the recipients have enjoyed face-to-face online meetings where ideas were discussed, shared, and expanded; conference calls where challenges and successes are reviewed; and continued correspondence of support during the COVID-19 pandemic.

Despite the challenges presented by the COVID-19 pandemic, the leaders of these LMIGs persevered. In many cases, the LMIGs thrived as the pandemic highlighted the importance of wellness, social connectedness, and self-care during these particularly stressful times. We look forward to seeing what these innovative LMIGs continue to develop throughout the remainder of the pandemic in the hopes that lifestyle medicine practices become the norm during this challenging time and beyond.
